# Sequential Comparison of Knee Muscle Strength after Anterior Cruciate Ligament Reconstruction between Hamstring Autograft and Tibialis Anterior Allograft: Propensity Score Matched Pair Analysis

**DOI:** 10.3390/diagnostics14141478

**Published:** 2024-07-10

**Authors:** Se-Han Jung, Chong Hyuk Choi, Sung-Hwan Kim, Kwangho Chung, Hyun-Soo Moon, Woongseob Sim, Min Jung

**Affiliations:** 1 Arthroscopy and Joint Research Institute, Yonsei University College of Medicine, Seoul 03722, Republic of Korea; drshjung@naver.com (S.-H.J.); choi8422@yuhs.ac (C.H.C.); orthohwan@gmail.com (S.-H.K.); khchung85@yuhs.ac (K.C.); oshsdesu@gmail.com (H.-S.M.); 2 Department of Orthopedic Surgery, Gangnam Severance Hospital, Yonsei University College of Medicine, Seoul 06273, Republic of Korea; 3 Department of Orthopedic Surgery, Severance Hospital, Yonsei University College of Medicine, Seoul 03722, Republic of Korea; aja0729@yuhs.ac; 4 Department of Orthopedic Surgery, Yongin Severance Hospital, Yonsei University College of Medicine, Yongin 16995, Republic of Korea

**Keywords:** anterior cruciate ligament reconstruction, graft choice, hamstring autograft, allograft, muscle strength, isokinetic testing

## Abstract

Among the graft options for anterior cruciate ligament reconstruction (ACLR), hamstring autografts are widely regarded as the preferred choice for primary ACLR among orthopedic surgeons worldwide. However, concerns persist regarding postoperative knee flexor weakness. We aimed to compare knee extensor and flexor strengths between hamstring autograft and tibialis anterior allograft groups in ACLR patients, who were propensity score-matched based on baseline characteristics. A retrospective analysis included 58 matched pairs who underwent isokinetic strength tests at 6 and 12 months post operation. Isokinetic muscle strength tests found no significant difference in knee extensor and flexor strength at 6 months post operation between the hamstring autograft and tibial anterior allograft groups. At 12 months, the hamstring autograft group exhibited significantly greater knee flexor deficit (total work and average power) compared to the allograft group, despite no differences in extensor strength or patient-reported outcomes. This study highlights the impact of hamstring autograft harvesting on muscle strength and recovery following ACLR in short-term period.

## 1. Introduction

The anterior cruciate ligament (ACL) is the knee ligament most frequently injured, particularly during sports activities [1]. In the general population, the age- and sex-adjusted annual incidence of ACL injuries is reported to be 68.6 per 100,000 person years [2]. Over time, the rate of ACL reconstruction (ACLR) has significantly increased as the primary surgical option for patients with significant instability following ACL injury [2,3,4,5,6,7,8]. Various graft options are currently utilized for ACLR [9]. There have been reports that allografts are associated with a higher graft re-rupture rate compared to autografts in young and active patients undergoing ACLR [10]. However, recent studies have generally shown comparable clinical outcomes and knee stability between autografts and allografts [11,12]. Therefore, both autografts and allografts are currently widely used in ACLR.

Among autograft options, the hamstring autograft, which involves the semitendinosus and gracilis tendons, is one of the most commonly used grafts worldwide [13,14,15]. However, each autograft option has associated donor site morbidity depending on the harvest site, and postoperative knee flexion weakness has been reported to be a significant concern following hamstring autograft harvest [16].

Strength deficits are commonly reported but are typically transient and resolve over time [17,18]. A randomized controlled study observed significant weakness at 3 months, which resolved by 6 months [17]. Despite this, several studies have demonstrated a persistent knee flexor peak torque deficit after hamstring autograft [1,19,20,21,22].

Extensor and flexor muscle weakness can occur not only after ACLR but also following various knee surgeries. Therefore, setting an appropriate control group to evaluate the specific effects of hamstring autograft alone is crucial. In this context, using the allograft group without any donor site harvest as a control group could be suitable. Some limited studies have compared muscle strengths between hamstring autograft and allograft [19,20]. According to these previous studies, more significant and prolonged knee flexor deficit were observed in the hamstring autograft group compared to the allograft group [19,20]. However, these studies did not match baseline characteristics to minimize the confounding factors, and there was no sequential comparison of changes in muscle strength over time after surgery.

This study aims to compare knee extensor and flexor strengths sequentially after ACLR between the hamstring autograft group and the tibialis anterior allograft group, with groups matched based on strict baseline characteristics. Rehabilitation after ACLR was standardized for all patients. A hinged brace was utilized for six weeks, during which the range of motion and weight-bearing was gradually increased. After six weeks, patients progressed to full weight-bearing without a hinged brace, gradually engaging in muscle strengthening exercises, and eventually returning to sports at the appropriate time. Education was provided to ensure patients could perform the rehabilitation protocols correctly. We hypothesized that hamstring autograft harvest would negatively affect knee flexor strength after ACLR.

## 2. Materials and Methods

### 2.1. Patients

**Figure 1:**
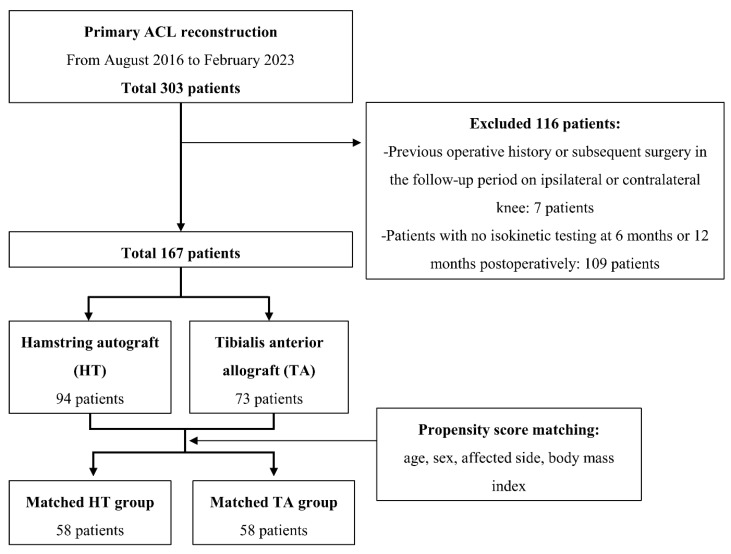
Patient flow diagram.

The medical records of the patients who underwent primary ACLR between August 2016 and February 2023 were retrospectively reviewed in this study. Inclusion criteria were as follows: (1) age older than 18 years old, (2) primary ACLR using either quadruple hamstring autograft or tibialis anterior allograft, and (3) completion of isokinetic quadriceps and hamstring strength testing at both 6 months and 12 months post operation. Exclusion criteria were: (1) primary ACLR using other autograft options (e.g., bone-patellar tendon-bone autograft), (2) concomitant ligament injuries, (3) previous surgical or traumatic history on the ipsilateral limb, and (4) history of surgical treatment or injury on the contralateral knee. After the application of inclusion and exclusion criteria, a total of 167 patients were included in the present study. And then A 1:1 matched group was formed to directly compare patients who underwent ACLR with hamstring autograft (HT group) versus tibialis anterior allograft (TA group). Propensity score matching was performed using age, gender, body mass index (BMI), and the affected side (right or left) as matching factors. Each group was allocated 58 patients (Figure 1). Our institutional review board reviewed and approved this study. Due to its retrospective nature and minimal risk, patient consent was waived with the board’s approval.

### 2.2. Surgical Procedures and Rehabilitation

Before the surgery, we explained the advantages and disadvantages of the available grafts for ACLR to the patient and discussed with them to decide on the graft to be used in the surgery. The surgical procedures for ACLR, including graft harvest, were performed by a single surgeon at a single institution. The hamstring autograft harvest was conducted before the arthroscopic procedures. The semitendinosus and gracilis tendons were identified near their distal attachments. Any accessory bands along each tendon were identified and trimmed. Using an open-loop tendon stripper, the tendons were divided up to the proximal musculotendinous junction. Subsequently, the distal attachments were detached from the tibia at the pes anserinus, and the final semitendinosus-gracilis graft was obtained. The obtained hamstring autograft was whipstitched using a No. 1 Ethibond (Ethicon, Inc., Cincinnati, OH, USA) at each end, passed through the loop of the Endobutton CL Ultra (Smith&Nephew, Watford, England, UK), and folded in half to create a four-stranded configuration. For tibialis anterior allografts, low-dose gamma-radiated fresh frozen allografts were used, which were 9–10 mm in diameter and 260–300 mm in length. In the same manner as the hamstring autograft, both ends of the allograft were whipstitched and used in a double-stranded configuration.

Thereafter, an arthroscopic procedure was conducted using an anterolateral portal. If meniscal lesions were identified, appropriate surgical procedures were performed before proceeding with the ACLR. The ACLR was carried out using the previously described methods [5]. The ACL was reconstructed at the anatomic footprints using the anteromedial portal technique. A suspensory fixator (Endobutton CL Ultra, Smith&Nephew, Watford, England, UK) was utilized for femoral fixation. Tibial fixation was achieved using a bioabsorbable interference screw, supplemented with post-tie fixation using a washer screw.

Following the surgical procedures, patients were instructed to initiate crutch-assisted partial weight-bearing ambulation. The range of motion was controlled using an adjustable hinged knee brace for 6 weeks, with a gradual increase in range of motion over time. Rehabilitation included a range of motion exercises and isometric quadriceps strengthening during the early postoperative period. By 6 weeks post operation, patients were cleared for full weight-bearing ambulation and began closed kinetic chain exercises. At 6 months post operation, patients were permitted to engage in activities such as jogging, swimming, and unrestricted muscle-strengthening exercises. Sports activities involving pivoting, jumping, or side-stepping were allowed at 9 months post operation.

### 2.3. Isokinetic Muscle Strength Testing

The Cybex isokinetic muscle test was performed at 6-month and 12-month follow-ups to measure extensor (quadriceps) and flexor (hamstring) isokinetic muscle strength using the Cybex isokinetic dynamometer (HUMAC NORM isokinetic machine, CSMI, Stoughton, MA, USA). Each patient was positioned on the examination table with a chair-back angle of 85° and their upper body and thighs were fixed with straps. The Cybex workout axis was aligned with the knee flexion axis, and the lower-extremity axis was parallel to the dynamometer arm. Patients were allowed to perform several repetitions to familiarize themselves with the test and received verbal instructions for the proper execution of the tests. Isokinetic muscle strength was recorded at 60°/s and 180°/s for knee extension and flexion during the exercise repetitions. Measurements for extensor and flexor peak torque (N·m), total work (N·m), and average power (Watts) were obtained. The limb symmetry index (LSI) was calculated for each value (peak torque, total work, average power).

### 2.4. Clinical Outcome Assessment

The clinical assessments of patient reported-outcome measures (PROMs) and knee stability related to ACL instability were conducted preoperatively and at 1 year post operation. PROMs included the pain visual analogue scale (VAS), Lysholm knee score, and International Knee Documentation Committee (IKDC) subjective score. Preinjury and postoperative activity levels were evaluated using the Tegner Activity Scale. Return to activity was defined as achieving the same Tegner scale level as before the injury, while near return to activity was defined as reaching one level lower. Anterior and rotational instability of the ACL were assessed using Lachman and pivot shift tests, performed by a single senior orthopedic surgeon for consistency.

### 2.5. Statistical Method

Propensity score matching was conducted to minimize potential bias and confounding when comparing the two groups (HT group vs. TA group). The factors selected for matching included age, sex, BMI, and affected side. Propensity score matching was performed with a caliper of 0.2 standard deviations. The balance of the matched groups was assessed using absolute standardized mean differences (ASMD), with all values <0.1 indicating a well-balanced matched group. The propensity score matching process was conducted using SAS software (version 9.4, SAS Inc., Cary, NC, USA).

The primary outcome measures of this study were the LSIs of the parameters representing the extensor and flexor isokinetic muscle strength. The secondary outcomes were PROMs, including the pain VAS, Lysholm knee score, and IKDC subjective score. Continuous variables were presented as mean ± standard deviation or median and interquartile range according to the normality test (Shapiro–Wilk test). Categorical variables were presented as count and percentage. To compare the overall baseline characteristics between the groups before propensity score matching, continuous variables were analyzed with an independent t-test or Mann–Whitney U test according to the normality of the data, and categorical variables were analyzed using the chi-squared test. For the comparison of variables between the matched group, a paired t-test or Wilcoxon signed-rank test was utilized for continuous variables, and McNemar’s test was utilized for categorical variables. Statistical analyses were performed using SPSS version 26.0 (IBM, Armonk, NY, USA), with statistical significance defined as *p* < 0.05. The required minimum sample size for this study was determined using a power analysis with G-power (version 3.1, Heinrich Hein University, Dusseldorf, Germany) to ensure adequate power to detect a significant difference. Given that the primary endpoint was the LSI of the isokinetic muscle strength, especially focusing on flexor strength, the mean difference and standard deviations of the LSI of the flexor peak torque from a previous study were selected [19]. To achieve a power of 80% and a two-sided alpha level of 0.05, the required minimum sample size was 44 for each group. Statistical power and the effect size for the study’s main significant data was also calculated.

## 3. Results

Overall data and matched data of the baseline characteristics of the patients are presented in Table 1. ASMD showed a well-balanced matched group. As a result of propensity score matching, a matched pair of 58 patients who underwent ACLR using hamstring autograft (HT group) and tibialis anterior allograft (TA group) were formed for analysis (Figure 1).

Sequential results from the Cybex test at 6 months and 12 months post operation were presented in Table 2. No significant differences were observed in parameters associated with extensor and flexor muscle strength between the groups at 6 months post operation. However, at 1 year post operation, the total work LSI of the flexor muscle during isokinetic exercise at both 60°/s and 180°/s was significantly lower in the HT group compared to TA group (at 60°/s, 74.4 ± 19.6 vs. 84.2 ± 24.8, *p* = 0.020; at 180°/s, 78.1 ± 19.2 vs. 85.8 ± 23.6, *p* = 0.027). Similarly, the average power LSI of the flexor muscle at 60°/s and 180°/s was significantly lower in the HT group at 1 year post operation (at 60°/s, 76.3 ± 17.3 vs. 85.3 ± 21.5, *p* = 0.029; at 180°/s, 78.1 ± 19.2 vs. 87.1 ± 22.1, *p* = 0.045). No significant differences were observed in parameters associated with extensor muscle strength between the groups. The power analysis indicated a power range of 93.7% to 97.7% for detecting statistically significant results in the muscle power comparison. The calculated effect sizes ranged from 0.407 to 0.461 for the significant results, indicating moderate practical differences in flexor muscle strength between the two groups.

Additionally, the assessments of ACL instability, including the Lachman test and pivot shift test, demonstrated no significant differences between the groups. PROMs including the VAS, Lysholm knee score, and IKDC subjective scores also showed no significant differences between the two groups. Furthermore, the return to activity, as assessed by the Tegner Activity Scale, did not differ between the groups (Table 3).

## 4. Discussion

The principal finding of this study is that at the 1-year follow-up Cybex test, the HT group exhibited a significantly greater flexor deficit compared to the TA group, despite no difference in flexor deficit between the groups at the 6-month follow-up after ACLR. Regarding extensor deficit, no significant deficits were noted between the groups at either the 6-month or 1-year follow-ups. These results suggest that both groups experienced overall extensor and flexor muscle deficits in the early stages after surgery; however, the recovery from flexor deficit was more insufficient in the HT group during the first year post operation.

Hamstring autograft is widely regarded as the preferred choice for primary ACLR among orthopedic surgeons globally. In a recent survey of 2130 sports medicine specialists, hamstring autograft accounted for 80.3% of the preferred graft choice for primary ACLR, being the most favored graft type on most continents except North America [13]. The hamstring autograft offers several advantages, including technical simplicity and fewer postoperative complications such as anterior knee pain, while ensuring comparable clinical outcomes and knee function compared to other autografts, such as the bone–patellar tendon–bone (BPTB) autograft [23]. However, it has been reported that hamstring autograft harvest can sometimes lead to flexion muscle weakness as a potential drawback. Konrath et al. [22] reported substantially altered muscle–tendon properties after hamstring autograft harvesting, resulting in knee flexor weakness. In contrast, Ardern et al. [21] found that hamstring autografts are less likely to significantly affect postoperative hamstring strength in athletes returning to sports. Notably, there is a scarcity of well-designed studies on this topic, and no consensus has been reached regarding the impact of hamstring autograft harvesting on muscle strength post operation.

Postoperative muscle strength comparisons among different autograft types have been extensively studied [1,19,20,21,24,25,26]. Sinding et al. [1], in a randomized controlled trial, reported that hamstring autograft harvesting led to impairments in both knee extensor and flexor strength, whereas quadriceps tendon autograft resulted in more pronounced impairments in knee extensor strength only. In a comparison between the BPTB and the hamstring autograft by Cristiani et al. [24], the BPTB group exhibited inferior quadriceps strength and poorer single-leg-hop test performance, while the hamstring groups exhibited inferior hamstring strength. Additionally, a systematic review and meta-analysis reviewing 14 related studies reported that the BPTB group showed extensor weakness whereas the hamstring group showed flexor weakness [26]. Based on these findings, it seems reasonable to assume that hamstring autograft harvesting may cause some degree of hamstring muscle weakness post operation.

Thigh muscle weakness is a common complication following various type of knee surgeries, including ACLR using different graft types [27,28,29]. Therefore, to specifically analyze the effect of hamstring autograft harvest on muscle strength, it is considered reasonable to compare muscle strength between the hamstring autograft group and an allograft group, where no harvest was performed, and all other procedures were conducted identically. This comparison provides an appropriate control group. To date, two studies have employed this approach [19,20]. Landes et al. [20] observed persistent knee flexor deficits with hamstring autografts but not with allografts. Similarly, Kim et al. [19] noted significant knee flexor weakness in both autograft and allograft groups, with a greater deficit in hamstring autograft group. They also reported similar clinical and functional outcomes between the groups, suggesting comparable overall results despite muscle strength differences [19]. The results of our study align with these findings. At the 1-year follow-up, the HT group exhibited a greater flexor deficit than the TA group, despite no significant differences in flexor deficit at the 6-month follow-up and postoperative clinical outcomes at 1-year follow-up between the groups. In this study, not only peak torque but also total work carried out and average power per repetition were evaluated. Of these three values, the two groups showed no difference in peak torque, but there was a significant difference in total work and average power. Total work is the more ‘functional’ measure of muscle performance, as work is torque sustained over distance in isokinetic strength testing. Average power reflects how effectively the muscle can perform work over time. This result indicates that the hamstring autograft group’s flexor muscle is functionally weaker and less effective than the allograft group at 1-year follow-up. Another notable aspect, unlike the two previous studies, is that the present study employed propensity score matching to form matched pairs between the comparison groups. The two previous studies did not match baseline characteristics between the groups. Therefore, findings of the present study provide a novel contribution by conducting a comparison after forming matched pairs through strict propensity score matching.

This study has several limitations that should be acknowledged. First, this study includes a short-term follow-up, as isokinetic testing was performed only up to the first year according to the current postoperative protocol in this cohort. Second, although guidelines for rehabilitation were provided to patients, they were not directly assisted in their rehabilitation, and thus detailed information regarding individual patients’ rehabilitation processes was not assessable. This lack of detailed rehabilitation data could introduce potential selection bias, as patient adherence and variability in rehabilitation efforts could impact the outcomes. Third, muscle strength was evaluated using only one modality. However, we employed isokinetic strength testing, which is currently the most objective and accurate method used in clinical settings. Given that this is a retrospective study, we had to apply the tests that were actually used in clinical settings to our study. Lastly, the effects of the significant primary results of this study were calculated to be moderate in size. The differences between the groups derived from this study should be carefully re-evaluated in clinical settings to determine whether they translate into clinically significant differences.

## 5. Conclusions

Isokinetic muscle strength tests revealed no significant differences in knee extensor and flexor strength between the hamstring autograft and tibialis anterior allograft groups at 6 months post operation. However, at 12 months post operation, the hamstring autograft group exhibited a greater knee flexor deficit compared to the allograft group, indicating less recovery of knee flexor strength in hamstring autograft group in the first year after anterior cruciate ligament reconstruction.

## Figures and Tables

**Table 1:** Baseline characteristics (before vs. after propensity score matching).

	Overall				Matched			
Variables	HT (*n* = 73)	TA (*n* = 94)	*p*-Value	ASMD	HT (*n* = 58)	TA (*n* = 58)	*p*-Value	ASMD
Age	23.0 (20.0, 33.0)	26.0 (20.0, 38.0)	0.300	0.194	23.00 (20.0, 35.0)	24.00 (19.0, 34.0)	0.598	0.006
BMI	24.11 (22.55, 25.96)	24.70 (22.39, 27.39)	0.309	0.190	24.39 (22.6, 26.2)	24.47 (22.4, 26.8)	0.474	0.026
Sex			0.012	0.405			>0.999	<0.001
Male	62 (84.9)	64 (68.1)			49 (84.5)	49 (84.5)		
Female	11 (15.1)	30 (31.9)			9 (15.5)	9 (15.5)		
Affected side			0.718	0.056			0.695	0.069
Right	36 (49.3)	49 (52.1)			28 (48.3)	30 (51.7)		
Left	37 (50.7)	45 (47.9)			30 (51.7)	28 (48.3)		

HT, hamstring autograft; TA, tibialis anterior allograft; ASMD, absolute standardized mean difference; BMI, body mass index.

**Table 2:** Comparison of Limb Symmetry Indices (LSI) between the groups.

	6 Months Post Operation				12 Months Post Operation			
Variables	HT Group	TA Group	*p*-Value	Effect Size	HT Group	TA Group	*p*-Value	Effect Size
Extensor 60°/s								
Peak torque LSI	67.4 ± 22.8	64.6 ± 22.1	0.468	0.124	80.3 ± 20.4	75.6 ± 18.8	0.168	0.240
Total work LSI	65.6 ± 22.1	65.6 ± 22.8	0.997	0.001	79.8 ± 19.3	77.3 ± 19.0	0.454	0.131
Average power LSI	67.9 ± 21.7	66.3 ± 18.3	0.945	0.080	79.0 ± 19.3	76.5 ± 18.4	0.470	0.133
Flexor 60°/s								
Peak torque LSI	71.6 ± 22.8	76.3 ± 21.9	0.210	0.210	80.6 ± 19.0	86.4 ± 19.3	0.116	0.303
Total work LSI	67.1 ± 25.9	67.5 ± 21.4	0.934	0.016	74.4 ± 19.6	84.2 ± 24.8	0.020 *	0.438
Average power LSI	72.1 ± 22.6	75.1 ± 23.3	0.400	0.131	76.3 ± 17.3	85.3 ± 21.5	0.029 *	0.461
Extensor 180°/s								
Peak torque LSI	70.0 ± 19.1	72.2 ± 20.3	0.546	0.112	82.0 ± 17.1	81.3 ± 20.7	0.840	0.037
Total work LSI	68.3 ± 20.4	70.7 ± 22.7	0.548	0.111	80.5 ± 17.3	82.0 ± 22.1	0.654	0.076
Average power LSI	67.2 ± 21.0	69.9 ± 21.6	0.481	0.127	81.2 ± 18.2	81.0 ± 22.2	0.947	0.010
Flexor 180°/s								
Peak torque LSI	79.1 ± 22.3	77.2 ± 19.3	0.632	0.091	84.1 ± 17.2	87.2 ± 17.1	0.246	0.181
Total work LSI	73.2 ± 26.0	71.9 ± 26.4	0.791	0.050	76.7 ± 21.1	85.8 ± 23.6	0.027 *	0.407
Average power LSI	74.2 ± 24.4	72.0 ± 25.1	0.655	0.089	78.1 ± 19.2	87.1 ± 22.1	0.045 *	0.435

* Statistical significance.

**Table 3:** Comparison of patient-reported outcome measures and knee stability at 1-year follow-up between HT and TA group.

Variables	HT Group	TA Group	*p*-Value	Effect Size
VAS	14.3 ± 17.6	11.4 ± 9.9	0.313	0.203
Lysholm knee score	84.9 ± 11.6	85.8 ± 14.8	0.737	0.068
IKDC subjective score	75.8 ± 17.3	74.7 ± 16.9	0.679	0.064
Preinjury Tegner activity score	6.0 ± 1.9	5.5 ± 2.0	0.150	0.256
Tegner activity score	3.4 ± 1.6	3.7 ± 1.8	0.303	0.176
Return to activity rate	11 (19.0)	10 (17.2)	1.000	
Near return to activity rate	23 (39.7)	20 (34.5)	0.701	
Lachman test grade, 0/1/2/3	34/19/5/0	29/22/7/0	0.519	
Pivot shift grade, 0/1/2/3	47/10/1/0	42/14/2/0	0.527	

VAS, visual analogue scale; IKDC, International Knee Documentation Committee.

## Data Availability

The data presented in this study are available on request from the corresponding author.

## References

[1] Sinding K.S., Nielsen T.G., Hvid L.G., Lind M., Dalgas U. (2020). Effects of autograft types on muscle strength and functional capacity in patients having anterior cruciate ligament reconstruction: A randomized controlled trial. Sports Med..

[2] Sanders T.L., Maradit Kremers H., Bryan A.J., Larson D.R., Dahm D.L., Levy B.A., Stuart M.J., Krych A.J. (2016). Incidence of anterior cruciate ligament tears and reconstruction: A 21-year population-based study. Am. J. Sports Med..

[3] Liukkonen R.J., Ponkilainen V.T., Reito A. (2022). Revision rates after primary acl reconstruction performed between 1969 and 2018: A systematic review and metaregression analysis. Orthop J. Sports Med..

[4] Sanders T.L., Pareek A., Hewett T.E., Levy B.A., Dahm D.L., Stuart M.J., Krych A.J. (2017). Long-term rate of graft failure after acl reconstruction: A geographic population cohort analysis. Knee Surg. Sports Traumatol. Arthrosc..

[5] Moon H.S., Choi C.H., Yoo J.H., Jung M., Lee T.H., Choi K.H., Kim S.H. (2021). The graft insertion length in the femoral tunnel during anterior cruciate ligament reconstruction with suspensory fixation and tibialis anterior allograft does not affect surgical outcomes but is negatively correlated with tunnel widening. Arthroscopy.

[6] Bourke H.E., Salmon L.J., Waller A., Patterson V., Pinczewski L.A. (2012). Survival of the anterior cruciate ligament graft and the contralateral acl at a minimum of 15 years. Am. J. Sports Med..

[7] Moon H.S., Choi C.H., Jung M., Yoo J.H., Kwon H.J., Hong Y.T., Kim S.H. (2024). Small intercondylar notch size is not associated with poor surgical outcomes of anatomical single-bundle anterior cruciate ligament reconstructions. Clin. Orthop. Surg..

[8] Yoon K.H., Lee S.M., Park J.Y., Lee H.S., Hwang S.H. (2024). A comparison of results in older, middle-aged, and younger patients after primary anterior cruciate ligament reconstruction: Minimum 10-year follow-up. Clin. Orthop. Surg..

[9] Arnold M.P., Calcei J.G., Vogel N., Magnussen R.A., Clatworthy M., Spalding T., Campbell J.D., Bergfeld J.A., Sherman S.L. (2021). Acl study group survey reveals the evolution of anterior cruciate ligament reconstruction graft choice over the past three decades. Knee Surg. Sports Traumatol. Arthrosc..

[10] Kaeding C.C., Pedroza A.D., Reinke E.K., Huston L.J., Spindler K.P. (2015). Risk factors and predictors of subsequent acl injury in either knee after acl reconstruction: Prospective analysis of 2488 primary acl reconstructions from the moon cohort. Am. J. Sports Med..

[11] Lamblin C.J., Waterman B.R., Lubowitz J.H. (2013). Anterior cruciate ligament reconstruction with autografts compared with non-irradiated, non-chemically treated allografts. Arthroscopy.

[12] Mariscalco M.W., Magnussen R.A., Mehta D., Hewett T.E., Flanigan D.C., Kaeding C.C. (2014). Autograft versus nonirradiated allograft tissue for anterior cruciate ligament reconstruction: A systematic review. Am. J. Sports Med..

[13] Tuca M., Valderrama I., Eriksson K., Tapasvi S. (2023). Current trends in anterior cruciate ligament surgery. A worldwide benchmark study. J. ISAKOS.

[14] Alomar A.Z., Baltow B., AlMogbil I. (2023). Effect of anteromedial portal location on femoral tunnel inclination, length, and location in hamstring autograft-based single-bundle anterior cruciate ligament reconstruction: A prospective study. Knee Surg. Relat. Res..

[15] Liau Z.Q.G., Ng M.S.P., Low S.S.E., Chin B.Z., Hui J.H.P., Kagda F.H.Y. (2024). A novel practical method to predict anterior cruciate ligament hamstring graft size using preoperative mri. Knee Surg. Relat. Res..

[16] Widner M., Dunleavy M., Lynch S. (2019). Outcomes following acl reconstruction based on graft type: Are all grafts equivalent?. Curr. Rev. Musculoskelet. Med..

[17] McRae S., Leiter J., McCormack R., Old J., MacDonald P. (2013). Ipsilateral versus contralateral hamstring grafts in anterior cruciate ligament reconstruction: A prospective randomized trial. Am. J. Sports Med..

[18] Hardy A., Casabianca L., Andrieu K., Baverel L., Noailles T. (2017). Complications following harvesting of patellar tendon or hamstring tendon grafts for anterior cruciate ligament reconstruction: Systematic review of literature. Orthop. Traumatol. Surg. Res..

[19] Kim J.G., Yang S.J., Lee Y.S., Shim J.C., Ra H.J., Choi J.Y. (2011). The effects of hamstring harvesting on outcomes in anterior cruciate ligament-reconstructed patients: A comparative study between hamstring-harvested and -unharvested patients. Arthroscopy.

[20] Landes S., Nyland J., Elmlinger B., Tillett E., Caborn D. (2010). Knee flexor strength after acl reconstruction: Comparison between hamstring autograft, tibialis anterior allograft, and non-injured controls. Knee Surg. Sports Traumatol. Arthrosc..

[21] Ardern C.L., Webster K.E., Taylor N.F., Feller J.A. (2010). Hamstring strength recovery after hamstring tendon harvest for anterior cruciate ligament reconstruction: A comparison between graft types. Arthroscopy.

[22] Konrath J.M., Vertullo C.J., Kennedy B.A., Bush H.S., Barrett R.S., Lloyd D.G. (2016). Morphologic characteristics and strength of the hamstring muscles remain altered at 2 years after use of a hamstring tendon graft in anterior cruciate ligament reconstruction. Am. J. Sports Med..

[23] Li S., Su W., Zhao J., Xu Y., Bo Z., Ding X., Wei Q. (2011). A meta-analysis of hamstring autografts versus bone-patellar tendon-bone autografts for reconstruction of the anterior cruciate ligament. Knee.

[24] Cristiani R., Mikkelsen C., Wange P., Olsson D., Stålman A., Engström B. (2021). Autograft type affects muscle strength and hop performance after acl reconstruction. A randomised controlled trial comparing patellar tendon and hamstring tendon autografts with standard or accelerated rehabilitation. Knee Surg. Sports Traumatol. Arthrosc..

[25] Johnston P.T., Feller J.A., McClelland J.A., Webster K.E. (2021). Strength deficits and flexion range of motion following primary anterior cruciate ligament reconstruction differ between quadriceps and hamstring autografts. J. ISAKOS.

[26] Xergia S.A., McClelland J.A., Kvist J., Vasiliadis H.S., Georgoulis A.D. (2011). The influence of graft choice on isokinetic muscle strength 4-24 months after anterior cruciate ligament reconstruction. Knee Surg. Sports Traumatol. Arthrosc..

[27] Ericsson Y.B., Roos E.M., Owman H., Dahlberg L.E. (2019). Association between thigh muscle strength four years after partial meniscectomy and radiographic features of osteoarthritis 11 years later. BMC Musculoskelet. Disord..

[28] Väistö O., Toivanen J., Kannus P., Järvinen M. (2007). Anterior knee pain and thigh muscle strength after intramedullary nailing of a tibial shaft fracture: An 8-year follow-up of 28 consecutive cases. J. Orthop. Trauma.

[29] Mizner R.L., Petterson S.C., Stevens J.E., Vandenborne K., Snyder-Mackler L. (2005). Early quadriceps strength loss after total knee arthroplasty. The contributions of muscle atrophy and failure of voluntary muscle activation. J. Bone Jt. Surg. Am..

